# Exploring management and environment effects on edge‐of‐field phosphorus losses with linear mixed models

**DOI:** 10.1002/jeq2.20662

**Published:** 2025-01-07

**Authors:** Kelsey M. Kruger, Anita M. Thompson, Qiang Li, Amber M. Radatz, Eric T. Cooley, Todd D. Stuntebeck, Christopher J. Winslow, Emily E. Oldfield, Matthew D. Ruark

**Affiliations:** ^1^ Department of Soil Science University of Wisconsin‐Madison Madison Wisconsin USA; ^2^ Department of Biological Systems Engineering University of Wisconsin‐Madison Madison Wisconsin USA; ^3^ University of Wisconsin‐Division of Extension Madison Wisconsin USA; ^4^ United States Geological Survey, Upper Midwest Water Science Center Middleton Wisconsin USA; ^5^ Ohio Sea Grant College Program The Ohio State University Columbus Ohio USA; ^6^ Environmental Defense Fund Washington District of Columbia USA

## Abstract

Evaluating how weather, farm management, and soil conditions impact phosphorus (P) loss from agricultural sites is essential for improving our waterways in agricultural watersheds. In this study, rainfall characteristics, manure application timing, tillage, surface condition, and soil test phosphorus (STP) were analyzed to determine their effects on total phosphorus (TP) and dissolved phosphorus (DP) loss using 125 site‐years of runoff data collected by the University of Wisconsin Discovery Farms and Discovery Farms Minnesota. Three linear mixed models (LMMs) were then used to evaluate the influence of those factors on TP and DP losses: (1) a model that included all runoff events, (2) manured sites only, and (3) precipitation events only. Results show that the timing of manure application relative to the timing of a runoff event only had a marginal association with P loads and concentrations, although the majority of the runoff events were collected after 10 days of manure application. Tillage was as influential factor, with greater DP loads and concentrations associated with no‐till, especially during frozen conditions. Fields in this study had high STP values, but the model results only showed positive associations between DP load and DP flow‐weighted mean concentration (FWMC) loss at the 0‐ to 15‐cm depth. The precipitation event LMM (which included precipitation characteristics) was the model that resulted in the largest *R* ^2^ value. While the predictive capacity of the LMMs was low, they did illuminate the relative importance of management and environmental variables on P loss, and can be used to guide future research on P loss in this region.

## Core Ideas

- Edge‐of‐field phosphorus losses are affected by more variables than can be included in a single controlled study.
- Linear mixed models can be used to evaluate the relative effects of management and environment on event P loss.
- Manure timing only had a marginal association with event P loss.
- Greater event‐dissolved P (DP) loss was associated with no‐till frozen conditions.
- Fields with greater soil test phosphorus (STP) were associated with greater event DP loss.

**Abbreviations**
CT: conventional tillage
DP: dissolved P
EOF: edge‐of‐field
FWMC: flow‐weighted mean concentration
LMM: linear mixed model
NT: no‐till
RT: reduced tillage
SHG: soil hydrologic group
STP: soil test phosphorus
TP: total P

## 1 INTRODUCTION

Phosphorus (P) is an essential nutrient for crops but is not abundantly found in soils without additions from fertilizer or manure. Manure can be applied to croplands to add P to the soil, reduce soil compaction, and reduce the need for manure storage structures (Srinivasan et al., 2006). However, excess manure application increases the risk of P transport from the site in runoff and into waterways, especially when unincorporated into the soil (Allen & Mallarino, 2008; Withers et al., 2001). Tools such as the Runoff Risk Advisory Forecast have been created to help farmers plan their manure applications by identifying periods during which the risk of runoff is high (Murumkar et al., 2018). Understanding the best time to apply manure and avoiding application before rainfall or snowmelt can reduce P loss, while still providing the benefits pertaining to manure applications. This is particularly important in cold climates where winter manure applications are common and high amounts of runoff occur during mid‐winter and spring thaws (Good et al., 2012; Srinivasan et al., 2006; Stuntebeck et al., 2011).

Evaluating the effectiveness of best management practices, such as the timing of manure application, reducing tillage, increasing surface cover, and monitoring soil test phosphorus (STP), is important to reduce runoff and P from agricultural sources. Studies have investigated the timing of manure applications and P loss from agricultural fields mostly through laboratory experiments and plot‐scale studies (Cherobim et al., 2017; Owens et al., 2011; Singh et al., 2020). Increasing the time between manure application and a runoff event allows for liquid manure to infiltrate the soil and can reduce P loss. In a laboratory rainfall simulation experiment, Cherobim et al. (2017) found that total P (TP) and dissolved P (DP) losses were higher when rainfall occurred 24 h after manure application compared to 7 days after manure application. Similarly, a plot‐scale study found that nutrient concentrations were higher in runoff events close to nutrient applications (Owens et al., 2011). Although these studies demonstrate that P loss does decline with time since manure application, more field‐scale studies are needed to further understand how the timing of manure applications combined with other management and site factors impacts P loss in runoff to improve farmers’ decision‐making process (Singh et al., 2020).

Many edge‐of‐field (EOF) studies have used factorial experimental designs on an annual or seasonal scale to understand how management practices impact nutrient losses (Aryal et al., 2018; Tomer et al., 2016; Van Esbroeck et al., 2016). These studies manipulate a few variables across two or three field sites to evaluate their impact on nutrient losses. An alternative approach to understanding environmental and management effects on EOF runoff is to utilize multilevel linear mixed models (LMMs), which have been used in ecological studies and are a form of multivariate analysis that can compare effect sizes among inputs variables across a wide range of conditions and that are continuous and categorical in nature. Because of this, LMM approaches can be helpful statistical methods in agricultural research to understand the impact of environmental and management practices on field‐scale runoff and P losses while accounting for the nonindependent nature of observations that may come from the same agricultural field in space and time (Eagle et al., 2017; Woltman et al., 2012).

Long‐term EOF monitoring is important in understanding how environmental and management factors influence P loss and, ultimately, water quality. Discovery Farms Wisconsin and Discovery Farms Minnesota have collected 125 site‐years of event‐based EOF surface runoff data across 22 fields (2004–2019). Previously, studies have analyzed this long‐term EOF data using nonparametric analysis based on ranked data in frozen conditions (Komiskey et al., 2011) and regression tree analysis on a seasonal basis (Zopp et al., 2019) to understand P losses. These approaches provide insight into how frozen conditions influence P runoff. They also highlight the need for further investigation into how management and weather conditions interact and impact P loss on an event basis. The goal of this study was to assess the relative importance of environmental and farm management factors on P loads and concentrations in EOF surface runoff using LMMs. The first objective was to determine the relationships between DP and TP and DP or TP with runoff across runoff types (rainfall, rainfall on frozen soil, and snowmelt). Three additional objectives were created based on our ability to use input variables to determine the relative effect of (1) management and site variables, (2) manure timing (quantified as days between the last manure application and the runoff event), and (3) rainfall event characteristics on P loss.

**Core Ideas**

- Edge‐of‐field phosphorus losses are affected by more variables than can be included in a single controlled study.
- Linear mixed models can be used to evaluate the relative effects of management and environment on event P loss.
- Manure timing only had a marginal association with event P loss.
- Greater event‐dissolved P (DP) loss was associated with no‐till frozen conditions.
- Fields with greater soil test phosphorus (STP) were associated with greater event DP loss.

## 2 MATERIALS AND METHODS

### 2.1 Site descriptions

**FIGURE 1:**
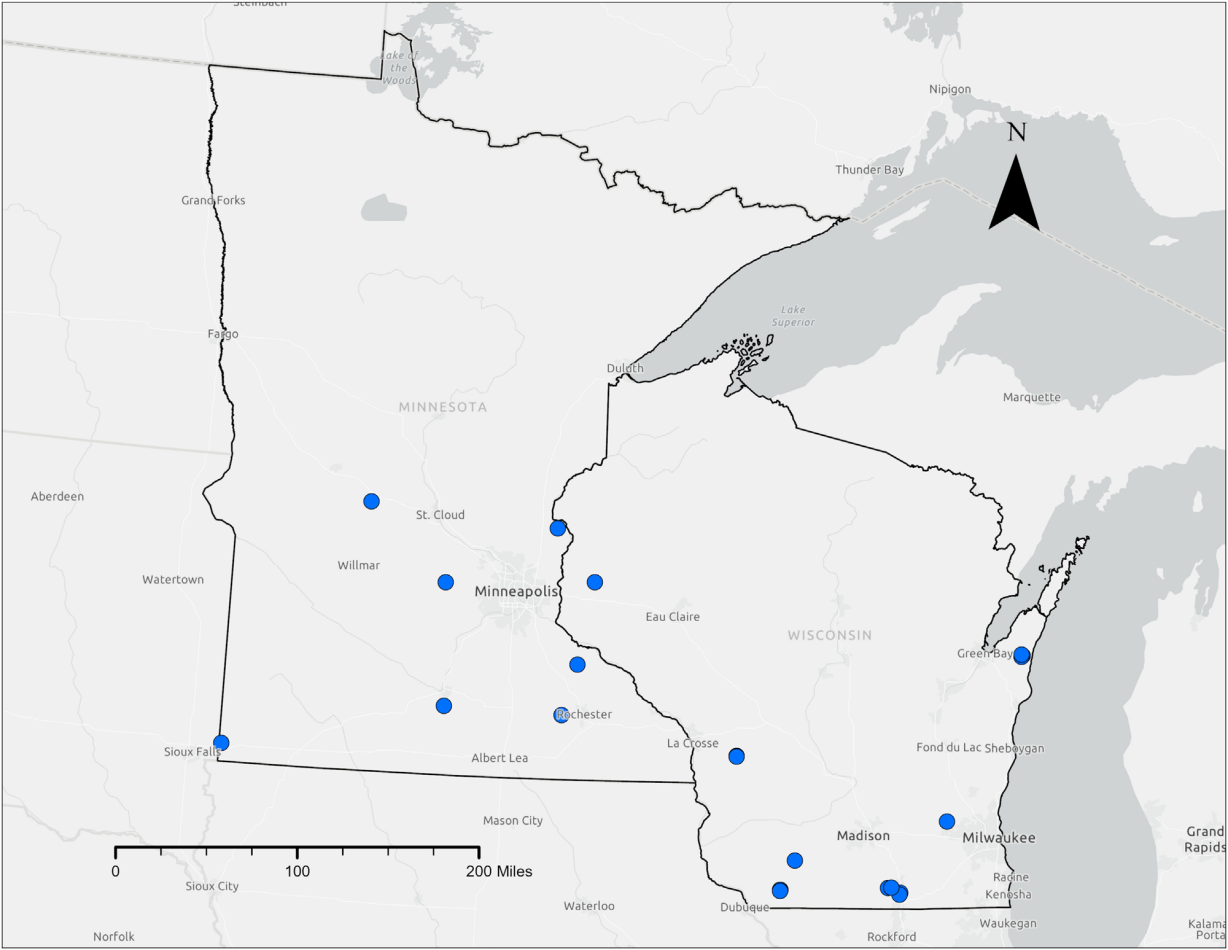
Map of Discovery Farms Sites in Wisconsin and Minnesota used in this study. Study sites within 5 km of each other are represented by one dot.

Discovery Farms Wisconsin and Discovery Farms Minnesota, in partnership with the US Geological Survey, have collected 125 site‐years (2004–2019) of event‐based EOF surface runoff data from 22 fields that contain a total of 1339 individual runoff events. Sixteen of the fields are in Wisconsin (*n* = 803 individual events) and six are in Minnesota (*n* = 536 events) (Figure 1). Across all sites, the dominant crops were corn, soybean, and alfalfa. The drainage area of these sites varied from 2.4 to 17.0 ha with an average of 7.9 ha. Most of these sites were located in fields with slopes >3% (19 sites), with only three sites having slopes ˂3%. Soil texture was characterized as primarily silt loam (17 sites) according to the National Cooperative Soil Survey (websoilsurvey.cs.egov.usda.gov), with other textures being loam (two sites), clay loam (one site), silty clay loam (one site), and sandy clay loam (one site).

### 2.2 Soil analysis and runoff sampling

Water quantity and quality data were collected at the edge of each field. Runoff events were defined as the time at which rainfall or snowmelt‐induced runoff began until the runoff ceased (Stuntebeck et al., 2008). Runoff (water quantity) was collected using H or trapezoidal flumes with plywood enclosures and wooden wingwalls in waterways or points of concentrated flow near the fields. Non‐submersible pressure transducers, along with nitrogen bubbler systems tracked the stage within the flumes. Water quality samples were collected through automated time‐based ISCO samplers and stored in a refrigerated enclosure (Stuntebeck et al., 2008; Rassmussen & Matteson, 2011). Up to 24 samples per day were collected from the ISCO samplers, which were combined into one flow‐weighted composite sample. These samples were retrieved within 24 h of the discharge and sent to the University of Wisconsin‐Stevens Point Water and Environmental Analysis Laboratory for analysis. The TP was analyzed following persulfate digestion (APHA, 2017) and DP was analyzed following filtration with a 0.45 µm filter; P concentration was determined using the ascorbic acid method (Murphy & Riley, 1962). Event runoff volume was multiplied by the concentration to obtain TP and DP loads for each collection time. If a single runoff event occurred over the course of two sampling days (which would include two separate TP and DP samples), the load for each day was summed and then divided by the total runoff across the sampling periods to calculate the flow‐weighted mean concentration (FWMC). Thus, all event concentrations are referred to as FWMC based on composite sampling within individual or across multiple sampling periods. All water collection, handling, and storage, as well as precipitation and soil temperature measurements are described in Stuntebeck et al., 2008 and Rassmussen and Matteson, 2011. Soil sampling for STP was conducted on each field at the onset of runoff monitoring at 0‐ to 5‐cm and 0‐ to 15‐cm depths and was determined using Bray‐P1 and Olsen methodology (Frank et al., 2012).

### 2.3 Dataset construction

Data was obtained from the United States Geological Survey (2021) and Komiskey et al. (2021). To investigate the mechanisms governing EOF runoff P losses, the dataset compiled for this study included event response variables (TP and DP loads and TP and DP FWMC), runoff amount (mm) and type, weather data, agronomic management practices, and site characteristics that were identified as having the potential to influence TP and DP loss. The runoff event type was categorized as either rainfall runoff on nonfrozen soil, rainfall runoff on frozen soil, or snowmelt runoff. The soil condition was considered frozen if the soil temperature was below 0°C at any point during a day. The weather data include individual rainfall event variables that were identified for each runoff event, including total precipitation, storm duration, average intensity, maximum intensity (as 5‐, 10‐, 30‐, and 60‐min maximum intensities), and antecedent rainfall 1, 2, and 3 days prior to the runoff event. Agronomic management was information collected from each farmer and includes if the site was tile drained or not (Y/N), the type of tillage used (no‐till [NT], reduced tillage [RT] [which includes vertical or strip tillage or tillage every other year], or conventional tillage [CT] [where at least one tillage pass occurred each year]), if the site had manure application during the monitoring time period (Y/N), and the active crop grown or most recently harvested (corn, alfalfa, corn and alfalfa, soybean, or pea). We also created a variable of “time since manure,” which was the days between a manure application and the runoff event. Site characteristics include soil hydrologic group (SHG), soil condition, and surface condition: 0–5 cm STP and 0–15 cm STP. SHG (A, B, C, and D) was obtained from Web Soil Survey (websoilsurvey.nrcs.usda.gov) and indicates the runoff potential of the soil and integrates effects of saturated hydraulic conductivity (to the least transmissive layer), depth to water‐impermeable layer, and depth to water table (NRCS, 2009). The soil condition is if the soil was frozen or not at the time of the runoff event. The surface condition variable described the state of the field at the time of the runoff event and included: (1) an actively growing crop (crop), (2) crop residue (no actively growing crop or a cover crop) (residue), and (3) no cover (no cover). Surface condition was designated as “crop” during the time between canopy closure of the current crop and a subsequent tillage pass or the harvest of corn silage, as this represents high potential for the canopy to reduce raindrop impact and subsequent soil displacement. For corn, the estimated canopy closure date was June 20th; for soy and pea, this date was July 15th; and for alfalfa, this date was June 20th. When there were multiple crops planted at the site, “crop” would start when the crop planted on over 50% of the site reached canopy closure. The residue category represented time periods when plant matter was left on the field without any tillage occurring or when cover crops were actively growing or recently terminated. The no‐cover category represented when the field was fallow, either following a tillage pass or the harvest of corn silage.

It is important to note that not all input variables in the model reflect the same number of runoff events. For example, five of the variables did not change over time for each site: SHG, tillage, manure application, 0–5 cm STP, and 0–15 cm STP, while four of the variables varied on an annual or within‐year basis: crop, surface condition, soil condition, and event type. All other variables were continuous variables that changed event by event.

### 2.4 Statistical analysis

The first analysis explored the relationships between DP and TP loss (as load and FWMC) for each runoff event type (rainfall on nonfrozen soil, rainfall on frozen soil, and snowmelt), as well as the relationship between DP or TP and runoff amount (mm). This analysis was conducted using least squares linear regression for each runoff type and the whole dataset. Data were analyzed using R Studio version 4.0.3 (R Core Team, 2020). A log‐transformation, log (count + 0.0025), was used when TP, DP, TP FWMC, and DP FWMC were not normally distributed. The package “psych” was used for summary statistics of continuous variables (Revelle, 2021). The coefficient of variation was calculated by dividing the standard deviation by the mean. Regression analyses were performed using the “lm” function in base “R.” Detailed descriptions of categorical variables considered in this study are provided in Table S1, and univariate statistics for runoff are provided in Table S2.

The second analysis consisted of regression modeling constructed from LMM. Specifically, the package “lme4” was used for LMM analysis using the “lmer” function, which fit with a Gaussian error distribution (Bates et al., 2015). The variables for LMM were chosen under the philosophy described by Oldfield et al. (2019) and Hobbs and Hilborn (2006), where only factors with established mechanisms influencing P loss were investigated. Our model included site and month as random effects to account for the nonindependent nature of observations in space and time. Three models were created to understand what may be influencing P loss. The first model (Model 1) was designed to include the greatest number of observations. SHG, crop, tillage type, soil surface condition, manure application, STP 0–5 cm, STP 0–15 cm, and runoff type were all included in the first model as fixed effects. The second model (Model 2) was constructed to understand how the number of days from manure application influences P loss. The same variables from the first model were used, except the binary for manure application was replaced with a continuous variable representing the number of days since the manure application (thus only including sites that received manure applications). The third model (Model 3) was created to understand how continuous precipitation variables were influencing P loss. In addition to the variables used in Model 1, precipitation amount, storm duration, average intensity, 5‐min max intensity, and antecedent rainfall 2 days were included. For all model development, the square root of the variance inflation factors was used to determine collinearity among the fixed effects, with a value of <2 indicating low collinearity. This reduced some weather variables from being included in Model 3. It should be noted that STP 0–5 cm and STP 0–15 cm were strongly correlated, but we decided to keep both variables in the model as STP is a major factor in P loss. Standardized coefficients were used to compare the effect sizes of the factors. Using the method described in Gelman (2008), standardizing involved subtracting the mean of each variable and dividing it by two standard deviations, leaving a mean of 0 and a standard deviation of 0.5. This allowed comparison between continuous and binary variables to understand their relative importance.

The following categories with each variable were used as reference variables, which are collectively included as the intercept of the LMM: SHG = A, crop = corn, tillage = conventional tillage, surface condition = actively growing crop, manure application = none, and event = rainfall on nonfrozen soil. The intercept for each model represents the reference conditions against which the other factors are compared. Standardized coefficients are used to compare how those factors influence TP, DP, TP FWMC, and DP FWMC. Large absolute values of standardized coefficients show there is a larger effect size (i.e., magnitude of impact) of a given factor on the response variable compared to the other factors considered. A negative coefficient means a decrease in the response variable, whereas a positive coefficient indicates an increase in the response variable. Individual categories with a variable are interpreted as being more or less influential compared to the reference category within that variable (based on the absolute value of the standardized coefficient) and more emphasis is placed on this statistic than *p*‐values (Anderson, 2019; Bradford et al., 2021) when discussing influential variables or categories.

Following Oldfield et al. (2019) and Hobbs and Hilborn (2006), we did not carry out model selection other than reducing collinearity. Determining a causal relationship demands either careful experimental design (e.g., randomized experiment) or statistical techniques to account for confounding factors that were not controlled for given the observational nature of this dataset. The models created are attempting to account for those confounding effects to determine the relevant effect size (magnitude of impact) of variables of interest on an outcome. Therefore, we did not carry out model selection as our goal was to account for variations in influential factors known to impact P loss (this approach is explained in Bradford et al., 2021). To explore the effect of days since manure (in Model 2) and precipitation (Model 3), data visualization was conducted with these a priori variables of interest along with other variables of importance determined as a result of the model output. For data visualization, all other variables in the model were assigned as the reference variable (for categorical) or the mean (for continuous).

## 3 RESULTS

### 3.1 Total and dissolved phosphorus

**FIGURE 2:**
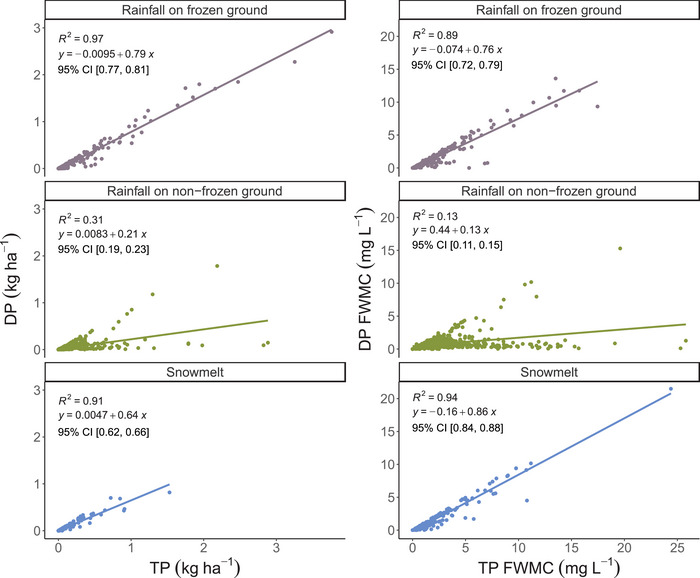
Relationships between event‐based total phosphorus load (TP) and dissolved phosphorus load (DP) (left) and event‐based TP flow‐weighted mean concentration (TP FWMC) and DP flow‐weighted mean concentration (DP FWMC) (right) without log transformation (*n* = 1339) analyzed by runoff type. Linear regression models, the 95% confidence interval (CI) of the slope, and *R* ^2^ were reported for each figure.

Event‐based TP loss ranged from 0 to 3.76 kg ha^−1^ with a median value of 0.02 kg ha^−1^ and TP FWMC ranged from 0 to 25.8 mg L^−1^ with a median value of 1.32 mg L^−1^. DP loss ranged from 0 to 2.91 kg ha^−1^ with a median of 0.01 kg ha^−1^ and DP FWMC ranged from 0 to 21.49 mg L^−1^ with a median of 0.62 mg L^−1^. All of these distributions were right‐skewed and displayed kurtosis (Table 1). Strong linear relationships were found between DP and TP, especially during snowmelt and runoff from rainfall on the frozen soil (*R* ^2^ values of 0.94 and 0.89 for FWMC and 0.91 and 0.97 for loads). Additionally, these events showed that DP was the dominant form of P, representing between 86% and 76% of FWMC and 64% and 79% of loads, for snowmelt and rainfall on frozen soil, respectively (Figure 2). Runoff on nonfrozen soil had lower *R* ^2^ for the relation between DP and TP FWMC and load (0.13 and 0.31, respectively) compared to other runoff types, and DP was only 13% and 21% of the TP FWMC and load based on the regression slopes (Figure 2). These results indicate a clear separation in the mechanisms controlling P loss during different event types. This can also be observed in Figure 2 as within the upper range of observed TP concentrations and loads, TP is almost entirely either DP or almost entirely PP.

**TABLE 1:** Univariate statistics for total phosphorus (TP), dissolved phosphorus (DP), and particulate phosphorus (PP) for loads and flow‐weighted mean concentrations (FWMC).

	Load			FWMC		
	TP (kg ha^−1^)	DP (kg ha^−1^)	PP (kg ha^−1^)	TP (mg L^−1^)	DP (mg L^−1^)	PP (mg L^−1^)
*n*	1339	1339	1339	1339	1339	1339
Minimum	0	0	0	0	0	0
First quartile	0.006	0.003	0.002	0.75	0.33	0.22
Mean	0.13	0.07	0.06	2.3	1.07	1.23
Median	0.02	0.01	0.01	1.32	0.62	0.48
Third quartile	0.12	0.04	0.04	2.71	1.06	1.23
Maximum	3.76	2.91	2.74	25.8	21.49	25.19
Std. dev	0.3	0.2	0.18	2.78	1.64	2.23
CV	2.31	2.86	16.67	1.21	1.53	1.81
Skewness	5.67	7.21	8.02	3.34	4.86	4.6
Kurtosis	43.81	68.1	90.97	15.94	34.42	29.86

Abbreviation: CV, coefficient of variation, Std. dev, standard deviation.

The flow‐flux relationship between runoff and TP had the greatest *R* ^2^ for runoff on nonfrozen soil (*R* ^2 ^= 0.74), although snowmelt and rainfall on frozen soil conditions had similar *R* ^2^ values (0.66 and 0.59, respectively). The flow‐flux relationship between runoff and DP followed this same trend, but resulted in slightly lower *R* ^2^ values. In all runoff conditions, the slope of the linear function between runoff and DP or TP was ˂1, indicating that there is a concentrating effect of greater DP and TP concentrations with increasing runoff amounts (Figure S1).

### 3.2 Linear mixed models

#### 3.2.1 Full dataset analysis (Model 1)

LMM results (*n* = 1318) showed that SHG, previous crop, and surface condition significantly influence load and FWMC for TP and DP (Table 2; Table S3). The hydrologic group was the most influential factor for TP load and FWMC, with groups B, C, and C/D having less P loss compared to group A (which has the lowest runoff potential). On average, alfalfa fields had less TP load and FWMC compared to corn fields (soybean fields also had less TP FWMC compared to corn). Events occurring with surface conditions of no cover and residue had greater TP loss and FMWC compared to when crops were actively growing. Rainfall events occurring on frozen soil had greater TP and DP load compared to events on nonfrozen soil. DP load and FWMC followed similar trends as TP for hydrologic group, crop, and soil condition. However, tillage was influential for DP load and FWMC, with NT and RT having greater loss compared to tilled fields (Table 2; Table S3). STP at 0–15 cm was positively associated with DP load and FWMC, while STP at 0–5 cm was negatively associated with DP load and FWMC.

**TABLE 2:** Standardized coefficients from linear mixed model one for total phosphorus (TP), dissolved phosphorus (DP), TP flow‐weighted mean concentrations (TP FWMC), and DP FWMC (*n* = 1318) (all data).

Variable	TP	DP	TP FWMC	DP FWMC
Intercept	**−1.08 ± 0.37**	**−1.88 ± 0.03**	**0.64 ± 0.29**	−0.21 ± 0.36
Hydrologic group B	**−0.63 ± 0.24**	*−0.40 ± 0.21*	**−0.42 ± 0.19**	−0.23 ± 0.23
Hydrologic group C	−0.47 ± 0.48	−0.40 ± 0.43	−0.43 ± 0.38	−0.26 ± 0.23
Hydrologic group C/D	**−0.67 ± 0.232**	**−0.44 ± 0.21**	**−0.51 ± 0.18**	−0.36 ± 0.22
Alfalfa	**−0.32 ± 0.07**	**−0.14 ± 0.06**	**−0.23 ± 0.05**	−0.08 ± 0.05
Corn and alfalfa	0.20 ± 0.23	0.21 ± 0.20	0.09 ± 0.18	0.14 ± 0.23
Pea	−0.05 ± 0.20	*0.33 ± 0.17*	−0.18 ± 0.13	*0.27 ± 0.14*
Soy	0.01 ± 0.05	0.02 ± 0.04	**−0.16 ± 0.03**	**−0.12 ± 0.04**
No‐till	0.08 ± 0.22	**0.47 ± 0.20**	0.13 ± 0.17	**0.58 ± 0.21**
Reduced tillage	−0.10 ± 0.3	0.32 ± 0.27	−0.13 ± 0.24	0.23 ± 0.29
No cover	**0.18 ± 0.06**	**0.11 ± 0.05**	**0.22 ± 0.04**	**0.15 ± 0.04**
Residue	**0.16 ± 0.07**	**0.15 ± 0.05**	**0.18 ± 0.04**	**0.18 ± 0.05**
Manure application	0.06 ± 0.27	0.17 ± 0.23	−0.10 ± 0.21	−0.04 ± 0.26
STP 0–15 cm	0.18 ± 0.17	0.22 ± 0.15	0.22 ± 0.14	*0.33 ± 0.17*
STP 0–5 cm	−0.17 ± 0.23	*−0.35 ± 0.20*	−0.12 ± 0.18	−0.37 ± 0.22
Snowmelt	−0.01 ± 0.07	*0.14 ± 0.05*	−0.04 ± 0.05	**0.14 ± 0.05**
Rainfall on frozen ground	**0.53 ± 0.07**	*0.61 ± 0.058*	−0.02 ± 0.05	**0.09 ± 0.05**
Marginal *R* ^2^	0.17	0.23	0.17	0.25
Conditional *R* ^2^	0.27	0.30	0.32	0.38

*Note*: All outcome variables were log‐transformed. Marginal *R* ^2^, describes variance from the fixed factors alone, and conditional *R* ^2^ describes variance from both fixed and random factors. Site and month were random factors. Manure application was coded as binary (0 = manure applied, 1 = no manure applied). Standardized coefficients allow for direct comparison between each variable. Intercept represents a site in hydrologic group A, planted with corn with an active crop growing, in nonfrozen conditions. Bolded values indicate that the associated *p* value was ≤ 0.05; italicized values indicate ≤ 0.1.

Abbreviation: STP, soil test phosphorus.

#### 3.2.2 Dataset with manure application and timing (Model 2)

Compared to Model 1, this dataset (*n* = 1040) only includes sites that had manure applications and thus also includes the input variable of manure timing (e.g., days since manure application). Model results for hydrologic group, surface condition, rainfall event, and current crop were similar to results in Model 1, although the relative influence of alfalfa was less in Model 2. STP 0–5 cm and STP 0–15 cm also followed the same trend as in Model 1, with STP 0–5 cm having an even greater negative standardized coefficient in Model 2 compared to Model 1. NT had the largest standardized coefficient for DP load and FWMC (with greater losses compared to tilled field); RT had negative standardized coefficients in Model 2, in contrast to results in Model 1 (Table 3). Days since manure application was an influential factor in the model for all variables, and was negatively associated with P loss (greater losses associated with fewer days occurring between manure application and a runoff event) (Table 3). For all response variables, *R* ^2^ values were similar to slightly greater in Model 2 compared to Model 1.

**TABLE 3:** Standardized coefficients from linear mixed model two for total phosphorus (TP), dissolved phosphorus (DP), TP flow‐weighted mean concentrations (TP FWMC), and DP FWMC (*n* = 1040).

Variable	TP	DP	TP FWMC	DP FWMC
Intercept	**−0.97 ± 0.18**	**−1.72 ± 0.17**	**0.58 ± 0.11**	−0.27 ± 0.17
Hydrologic group B	**−0.69 ± 0.18**	**−0.42 ± 0.18**	**−0.48 ± 0.10**	−0.23 ± 0.18
Hydrologic group C	−0.42 ± 0.37	−0.30 ± 0.38	−0.31 ± 0.2	−0.05 ± 0.39
Hydrologic group C/D	**−0.62 ± 0.17**	**−0.42 ± 0.17**	**−0.46 ± 0.1**	*−0.13 ± 0.18*
Alfalfa	−0.12 ± 0.08	−0.05 ± 0.07	−0.06 ± 0.05	−0.04 ± 0.06
Corn and alfalfa	0.18 ± 0.16	0.19 ± 0.17	0.09 ± 0.08	0.13 ± 0.18
Pea	0.01 ± 0.19	*0.31 ± 0.17*	−0.08 ± 0.14	*0.26 ± 0.15*
Soy	−0.07 ± 0.06	−0.04 ± 0.05	**−0.21 ± 0.04**	**−0.15 ± 0.04**
No‐till	0.24 ± 0.17	**0.66 ± 0.17**	**0.27 ± 0.10**	**0.76 ± 0.18**
Reduced‐tillage	−0.25 ± 0.25	0.17 ± 0.25	**−0.36 ± 0.13**	−0.05 ± 0.26
No cover	*0.11 ± 0.06*	*0.10 ± 0.05*	**0.14 ± 0.05**	**0.12 ± 0.05**
Residue	0.06 ± 0.07	0.10 ± 0.06	*0.10 ± 0.05*	**0.14 ± 0.05**
Days since manure application	**−0.30 ± 0.06**	**−0.24 ± 0.05**	**−0.20 ± 0.04**	**−0.20 ± 0.04**
STP 0–15 cm	0.07 ± 0.13	0.16 ± 0.14	*0.14 ± 0.07*	0.29 ± 0.14
STP 0–5 cm	−0.15 ± 0.19	**−0.55 ± 0.19**	−0.08 ± 0.10	**−0.5 ± 0.20**
Snowmelt	−0.08 ± 0.08	0.10 ± 0.07	−0.06 ± 0.03	**0.15 ± 0.06**
Rainfall on frozen soil	**0.48 ± 0.09**	**0.59 ± 0.07**	−0.05 ± 0.06	0.08 ± 0.06
Marginal *R* ^2^	0.21	0.29	0.21	0.32
Conditional *R* ^2^	0.30	0.36	0.31	0.39

*Note*: All the outcome variables in the table were log‐transformed. Marginal *R* ^2^, describes variance from the fixed factors alone, and conditional *R* ^2^, describes variance from both fixed and random factors, reported for each response variable. Site and month were random factors. Standardized coefficients allow for direct comparison between each variable. Intercept represents a site in hydrologic group A, planted with corn with an active crop growing, in nonfrozen conditions. Bolded values indicate that the associated *p* value was ≤ 0.05; italicized values indicate ≤ 0.1.

Abbreviation: STP, soil test phosphorus.

#### 3.2.3 Dataset with complete rainfall data (Model 3)

Compared to Model 1, this dataset (*n* = 947) includes only rainfall‐driven runoff events, but includes all weather data (e.g., precipitation, storm duration), as well as the categories of manure application (yes or no) and soil condition during rainfall (frozen or non‐frozen). The influence of hydrologic group, previous crop, surface condition, tillage, and event type was similar to Model 1. Larger TP and DP loads were associated with frozen soil compared to nonfrozen soil, and with manured sites compared to non‐manured sites. Precipitation had significant factors in the model for both TP and DP loads (with greater precipitation leading to greater loading), but not for FWMC (Table 4). Other rainfall characteristics, such as 5‐min maximum intensity of an event and antecedent rainfall 2 days prior to a rainfall event also demonstrated potential influence on TP and DP loads, with greater loads associated with higher intensity rainfall events and when there was a greater amount of rainfall 2 days prior to the rainfall event.

**TABLE 4:** Standardized coefficients from linear mixed model three for total phosphorus (TP), dissolved phosphorus (DP), TP flow‐weighted mean concentrations (TP FWMC), and DP FWMC (*n* = 973).

Variable	TP	DP	TP FWMC	DP FWMC
Intercept	**−0.39 ± 0.35**	**−1.23 ± 0.30**	**0.48 ± 0.21**	−0.08 ± 0.27
Hydrologic group B	**−0.61 ± 0.22**	*−0.37 ± 0.19*	**−0.42 ± 0.17**	−0.22 ± 0.23
Hydrologic group C	−0.37 ± 0.45	−0.31 ± 0.39	−0.32 ± 0.04	−0.13 ± 0.48
Hydrologic group C/D	**−0.62 ± 0.22**	*−0.36 ± 0.19*	**−0.53 ± 0.17**	−0.35 ± 0.22
Alfalfa	**−0.38 ± 0.08**	−0.13 ± 0.07	**−0.34 ± 0.05**	−0.09 ± 0.06
Corn and alfalfa	0.25 ± 0.21	0.26 ± 0.18	0.18 ± 0.17	0.23 ± 0.23
Pea	−0.27 ± 0.19	0.14 ± 0.16	*−0.24 ± 0.13*	0.20 ± 0.14
Soy	−0.06 ± 0.05	−0.04 ± 0.05	**−0.19 ± 0.04**	**−0.16 ± 0.04**
No‐till	0.05 ± 0.21	**0.55 ± 0.18**	0	**0.58 ± 0.21**
Reduced tillage	−0.27 ± 0.28	0.26 ± 0.24	−0.32 ± 0.22	0.12 ± 0.3
No cover	**0.19 ± 0.06**	*0.09 ± 0.05*	**0.22 ± 0.04**	**0.11 ± 0.04**
Residue	**0.16 ± 0.07**	**0.15 ± 0.06**	**0.22 ± 0.05**	**0.20 ± 0.05**
Manure application	−0.12 ± 0.24	0.27 ± 0.21	−0.15 ± 0.2	−0.08 ± 0.26
Nonfrozen soil	**−0.76 ± 0.08**	**−0.84 ± 0.06**	0.08 ± 0.05	*−0.10 ± 0.06*
STP 0–15 cm	0.24 ± 0.16	*0.25 ± 0.14*	**0.01 ± 0.003**	**0.01 ± 0.004**
STP 0–5 cm	−0.23 ± 0.21	**−0.44 ± 0.18**	0	*0*
Precipitation	**0.46 ± 0.06**	**0.44 ± 0.05**	0	0.06 ± 0.04
Storm duration	0.05 ± 0.05	0.06 ± 0.04	−0.05 ± 0.03	−0.02 ± 0.04
Average Intensity	−0.06 ± 0.05	−0.04 ± 0.04	−0.04 ± 0.03	−0.01 ± 0.04
5‐Min max intensity	**0.30 ± 0.06**	**0.12 ± 0.05**	**0.17 ± 0.04**	−0.01 ± 0.05
Antecedent rainfall of 2 days	**0.17 ± 0.04**	0.15 ± 0.03	−0.04 ± 0.03	−0.02 ± 0.03
Marginal *R* ^2^	0.33	0.38	0.26	0.29
Conditional *R* ^2^	0.42	0.45	0.39	0.44

*Note*: All the outcome variables in the table were log‐transformed. Marginal *R* ^2^ describes variance from the fixed factors alone, and conditional *R* ^2^ describes variance from both fixed and random factors, reported for each response variable. Site and month were random factors. Manure application was coded as binary (0 = manure applied, 1 = no manure applied); Soil condition was coded as binary (0 = nonfrozen soil; 1 = frozen soil). Standardized coefficients allow for direct comparison between each variable. Intercept represents a site in hydrologic group A, planted with corn with an active crop growing, in frozen conditions. Bolded values indicate that the associated *p* value was ≤ 0.05; italicized values indicate ≤ 0.1.

Abbreviation: STP, soil test phosphorus.

#### 3.2.4 Model visualization

After developing the models, a priori factors and those with large absolute unstandardized coefficients (Supporting Information) were chosen to visualize their relationships with response variables. Since there is a large degree of variability in this dataset, these model results indicate which factors might have the strongest influence on P loss. TP and DP FWMC were strongly influenced by tillage and surface condition in Model 2, and were selected along with days since manure application (the a priori variable of interest) for visualization. For Model 3, soil condition (frozen vs. non‐frozen), soil surface condition, and tillage practice were evaluated within the relationship between TP or DP and precipitation amount.

**FIGURE 3:**
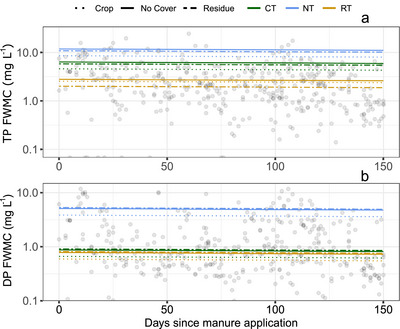
Modeled relationship (Model 2) between total phosphorus flow‐weighted mean concentration (TP FWMC) (a) or dissolved phosphorus flow‐weighted mean concentration (DP FWMC) (b) and the number of days since manure application. Each regression line is calculated from the unstandardized coefficients (Table S3) and isolates the potential effect of the days since manure application when the event occurs on TP and DP FWMC, for each combination of surface condition (crop, no cover, residue) and tillage (conventional tillage, CT; no tillage, NT; reduced tillage, RT). Individual observations are also plotted. We log‐transformed all response variables in our model, but show the modeled response as non‐transformed for ease of interpretation.

TP and DP FWMC were strongly influenced by tillage and surface condition. For TP and DP FWMC, NT had the greatest values, followed by CT, then RT, while CT and RT were similar for DP FWMC (Figure 3). Within all tillage categories, soil without cover had the highest TP and DP FWMC, followed closely by soil with crop residue; soils with an actively growing crop had the lowest FWMCs. However, no combination of tillage and surface soil condition appeared to lead to substantial change in DP or TP FWMC as the amount of time between manure application and the runoff event increased.

**FIGURE 4:**
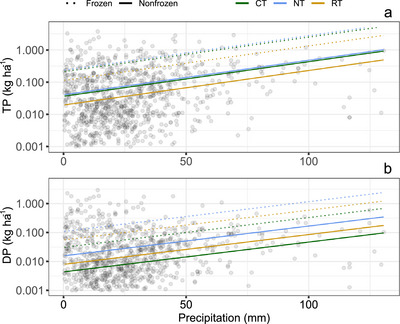
Modeled relationship (Model 3) between total phosphorus (TP) load (a) or dissolved phosphorus (DP) load (b) and precipitation associated with each runoff event. Each regression line is calculated from the unstandardized coefficients (Table S4) and isolates the potential effect of precipitation on TP and DP load, for each combination of soil condition (frozen or non‐frozen) and tillage (conventional tillage, CT; no tillage, NT; reduced tillage, RT). Individual observations are also plotted. We log‐transformed all response variables in our model, but show the modeled response as non‐transformed for ease of interpretation.

**FIGURE 5:**
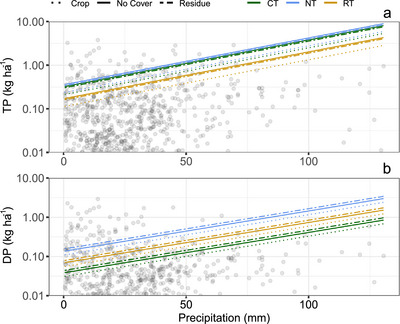
Modeled relationship (Model 3) between total phosphorus (TP) load (a) or dissolved phosphorus (DP) load (b) and precipitation associated with each runoff event. Each regression line is calculated from the unstandardized coefficients (Table S4) and isolates the potential effect of precipitation on TP and DP load, for each combination of surface condition (crop, no cover, residue) and tillage (conventional tillage, CT; no tillage, NT; reduced tillage, RT). Individual observations are also plotted. We log‐transformed all response variables in our model, but show the modeled response as non‐transformed for ease of interpretation.

Visualization of Model 3 demonstrates the overall positive relationship between precipitation and DP and TP loss (Figures 4 and 5). This relationship was consistent regardless of soil condition, surface condition, or tillage (i.e., slopes are all parallel in Figures 4 and 5). Frozen soil had consistently greater P loss compared to nonfrozen soil, but the influence of tillage varied between DP and TP. NT sites were associated with greater DP loss under each soil condition compared to other tillage practices, while NT and CT were similar with respect to TP loss (Figure 4). A similar trend with tillage was observed when compared across surface conditions (Figure 5). Surface condition had a less dramatic effect on P loss compared to soil condition (frozen vs. non‐frozen).

## 4 DISCUSSION

The type of runoff event (precipitation vs. snowmelt) had consistent differences in P loss, with DP representing most of the TP loss during snowmelt, but DP representing less than a quarter of the TP loss during rainfall on nonfrozen soil. However, there was not a consistent difference in the quantity of TP loss between snowmelt and precipitation on nonfrozen soil (Tables 2 and 3; Tables S3 and S4). Precipitation on frozen soil was similar to snowmelt, with most of the P loss occurring as DP, and consistently was associated with greater P loss compared to precipitation on nonfrozen soil (Tables 2 and 3; Table S3 and S4; Figure 4). This aligns with previous literature that describes an increase in DP load and concentration due to snowmelt and rainfall on frozen soil runoff (Hansen et al., 2000; Li et al., 2011; Panuska et al., 2008). Findings from Hoffman et al. (2019) showed that TP loss was primarily driven by rainfall on nonfrozen soil, and DP made up 74% and 85% of TP during snowmelt and rainfall on frozen soil runoff events, respectively. They attributed this to a lack of infiltration and soil particle mobilization. Similarly, greater amounts of DP and DP FWMC soon after manure application could be due to a lack of infiltration during frozen conditions (Stock et al., 2019). However, K. Liu et al. (2013) suggest that the volume of runoff, not the timing of manure, was the most important factor when assessing nutrient loss. Collectively, this highlights the continued need to address P loss reduction strategies when the soil is frozen.

This dataset revealed a limited direct interaction between the time between the manure application and the runoff event on P loss. Previous studies have shown that P losses increase when rainfall or snowmelt occurs soon after manure applications (Klausner et al., 1976; Komiskey et al., 2011; Vadas et al., 2011). In particular, winter manure applications have been shown to increase DP (Cherobim et al., 2017; Vadas et al., 2019). These studies indicate that increasing the time between manure applications and precipitation or snowmelt can help reduce P runoff. Across all events, our results do show a negative relationship between days since manure application and P loss, suggesting that higher P loss can occur close to a manure application, but overall, this relationship was not dramatic; only small differences were shown to occur over time. Model 2 results signify that whereas days since manure application is a significant factor, other factors such as soil condition, tillage, and STP were much more influential on the amount of P loss during a single runoff event. This dataset did have a limited number of events that occurred within the 0‐ to 10‐day period (which is the time period the Runoff Risk Advisory Forecast analyzes manure application risk, manureadvisorysystem.wi.gov) after a manure application (*n* = 23) relative to the total amount of events observed (*n* = 1040). Furthermore, 15 of these runoff events occurred in February and March, when snowmelt and precipitation begin to increase after the winter period. Overall, runoff events that occur closer to manure applications do have higher P loads and concentrations, and consideration of frozen or nonfrozen conditions when manure is applied is important for understanding these losses.

The type of tillage was influential for TP, DP, TP FWMC, and DP FWMC. NT was associated with the greatest P losses, specifically for DP and DP FWMC in frozen conditions. Our results are in stark contrast to K. Liu et al. (2014), which showed that transitioning from CT (minimal tillage except to redistribute residue) to RT (tillage only every second year) led to a decrease in DP FWMC by 46%, primarily occurring during snowmelt. Our results do align with previous studies that show greater P losses under NT (Daryanto et al., 2017; Singh et al., 2020; Zopp et al., 2019) and can be attributed to the lack of surface roughness when NT occurs, as well as an accumulation of P in the top layers of soil due to its low mobility (Hansen et al., 2000). In our dataset, the NT and RT sites had greater STP in the 0–5 cm (91.4 ± 36.3 ppm; 126.8 ± 31.7 ppm, respectively) compared to CT sites (50.4 ± 15.7 ppm) confounding our ability to separate the individual influence of tillage and STP. Model results showed NT as a significant factor with a strong association with DP, which was the dominant form of P loss.

STP content at 0‐ to 15‐cm depth was moderately and positively associated with all P loss measurements, while 0‐ to 5‐cm depth was negatively associated. This is partially in contrast to previous work on this dataset aggregated on a seasonal basis, showing a clear positive effect of both STP 0–5 and 0–15 cm with P loss (Zopp et al., 2019). Long‐term manure application, especially on NT fields, has been shown to cause elevated STP content, especially in the upper 5 cm of the soil (Andraski et al., 2003). Furthermore, STP has historically been considered an important variable in predicting P loss (P indices) and positive in relation. It has been suggested that sampling STP at both the 0‐ to 5‐cm and 0‐ to 15‐cm depths can provide better predictions of P loss from agricultural fields (Osterholz et al., 2020). The counterintuitive results in our study with STP 0–5 cm are likely a result of how the event‐based dataset is constructed; only one STP value exists per site and is associated with hundreds of events. This in turn highlights some drawbacks with event‐based water quality analysis; factors that are site‐dependent require a large number of sites to properly assess their effect.

The presence of a crop, residue, or lack of cover was associated with differences in P loss. The greatest amount of TP loss in this dataset was associated with the no‐cover stage, reflecting results from Plach et al. (2019), which points to the nongrowing season as the high‐risk period for P loss in cold climates. Model results show that the presence of a crop is associated with lower amounts of TP and DP loss (Figures 4 and 5). Having active crop growth can reduce the rate of runoff and provide a buffer to the soil, as the canopy can slow the impact of raindrops hitting the surface, thus reducing soil detachment and P loss (Ma et al., 2014). However, residue conditions showed similar results to those of no cover conditions and were also associated with greater P loss compared to an actively growing crop. Residue conditions represented a range of conditions in terms of residue biomass (e.g., corn residue vs. soybean residue), an actively growing cover crop, or both, and almost exclusively under NT. Thus, we would expect the residue association with greater P loss to be confounded with the clearer connection between NT and increased P loss. However, residue can also be a source of P, particularly DP, as the residue decomposes and runoff occurs (K. Liu et al., 2014; Messiga et al., 2010). Models 1 and 3 show an increase in DP load and DP FWMC in both residue and frozen conditions (Tables 2 and 4; Tables S3 and S5). This could imply that during freeze‐thaw cycles, there is elevated DP loss. Freeze‐thaw cycles can accelerate the decomposition process, increasing the amount of nutrients released from residue and transported during snowmelt or rainfall on frozen soil (Bechmann et al., 2005; J. Liu et al., 2019).

Other variables considered in the models, such as crop, led to varied levels of association. No single crop was influential across all models. Alfalfa was associated with the lowest P loss out of the crops in this study. This could possibly be due to the year‐round cover provided, and that prior to seeding, the sites are tilled. Modeled losses were also lower for alfalfa than the other crops; however, alfalfa was not always significant to P loss and did not always have a high influence in comparison to the other crops. An actively growing alfalfa crop also has confounding factors such as lack of tillage and manure application. Results from Young and Mutchler (1976) compared manure applications on NT alfalfa with fall‐tilled continuous corn and found that alfalfa had higher runoff and P loss. The authors suggest that manure application on rough plowing, rather than applying to a non‐tilled field, helps reduce runoff and P loss. All of the alfalfa fields in this study are tilled before seeding, possibly reducing the amount of P on the soil surface. The effect of crop type on P loss was not conclusive but rather points to the other management conditions that may lead to increased P runoff.

The Natural Resources Conservation Service (NRCS) SHG helps explain the runoff potential from a site, capturing the soil type and infiltration rate of a soil. These factors generally had a tight association with P loss in the models, indicating that the hydrologic group is important to understanding the risk of P loss at a particular site (Tables 2 and 4; Tables S3 and S5). However, our results are counterintuitive, as soils with greater runoff potential were associated with lower P losses. TP and DP loads were significant with several weather variables within Model 3 (Table 4; Table S5). Rainfall intensity is an important consideration with P transport in runoff (Shigaki et al., 2007). Multiple intensity variables were tested in the models, but the 5‐min max intensity improved both the marginal and conditional *R* ^2^ value and had the tightest association with P runoff compared to other models considered. Out of the weather characteristics considered (precipitation, storm duration, average intensity, 5‐min max intensity, and antecedent rainfall for 2 days), 5‐min max intensity had the second strongest association with TP and the strongest association with TP FWMC. High‐intensity rainfall can exceed infiltration capacities and displace soil, which is associated with increased particulate P loss (Fraser et al., 1999; Sporre‐Moeny et al., 2004). Likewise, antecedent rainfall was significant to P loads. More saturated soil may be at risk for more runoff, as the water stored in the soil peaks during the storm. Danz et al. (2013) described antecedent rainfall as one of the most important characteristics for TP loads, as it provides information on the amount of water in the watershed prior to the runoff event.

## 5 CONCLUSION

This study provides information on how the timing of manure applications, surface condition, tillage, soil condition, and weather conditions affect single‐event EOF P runoff in Wisconsin and Minnesota. First, this study shows that the timing of manure applications only has a marginal association with P loads and concentrations over long time periods (most of the data were collected after 10 days of manure application). Second, this research highlights the impact of NT on P loss. Greater DP loads and concentrations are observed at NT sites, especially during frozen conditions. Third, the sites in this study had high amounts of STP at both the 0‐ to 5‐cm and 0‐ to 15‐cm depths, but the model results only showed positive associations between DP and DP FWMC loss at the 0‐ to 15‐cm depth.

LMM methods can be applied to other unstructured agricultural datasets to understand which practices may have a close association with P loss. In particular, the models in this study had a wide range of *R* ^2^, showing high variability based on the factors included. For further improvement, incorporating both the number of days since manure application and weather variables, including snowmelt data, into a single model (as opposed to analyzing them separately) may increase the *R* ^2^ value. This would provide a deeper understanding of how these significant factors influence P runoff in cold climates. However, it is clear that continued monitoring is needed to provide a larger distribution of STP values, tillage practices, and soil characteristics, in order to improve our understanding of P losses and develop a highly predictive event‐based model.

## AUTHOR CONTRIBUTIONS

**Kelsey M. Kruger**: Conceptualization; data curation; formal analysis; investigation; methodology; visualization; writing—original draft; writing—review and editing. **Anita M. Thompson**: Conceptualization; funding acquisition; investigation; project administration; supervision; writing—review and editing. **Qiang Li**: Validation; writing—review and editing. **Amber M. Radatz**: Data curation; resources; writing—review and editing. **Eric T. Cooley**: Data curation; resources. **Todd D. Stuntebeck**: Funding acquisition; project administration; supervision; writing—review and editing. **Christopher J. Winslow**: Investigation; project administration; supervision; validation. **Emily E. Oldfield**: Formal analysis; methodology; validation; writing—review and editing. **Matthew D. Ruark**: Conceptualization; formal analysis; funding acquisition; project administration; supervision; validation; writing—review and editing.

## CONFLICT OF INTEREST STATEMENT

The authors declare no conflicts of interest.

## Supporting information



Supplementary materials include a table describing the number of sites in each category of each variable (Table S1), univariate statistics of runoff and sediments (which are additional response variables not presented in this study) (Table S2), and linear mixed model output as unstandardized coefficients (Table S3, S4, S5). In addition, the graphical representation of the flow‐flux relationship, presented on a runoff type basis is provided.

## Data Availability

Data used in this publication is published in Dryad: https://doi.org/10.5061/dryad.hx3ffbgpv.
