# Supplementary material for: Exploring management and environment effects on edge‐of‐field phosphorus losses with linear mixed models
Source: J Environ Qual. 2025 Jan 7;54(2):450–64. doi: 10.1002/jeq2.20662 (PMC11893287; doi:10.1002/jeq2.20662)
Supplement: Supplementary file 1 — Supplementary materials include a table describing the number of sites in each category of each variable (Table S1), univariate statistics of runoff and sediments (which are additional response variables not presented in this study) (Table S2), and linear mixed model output as unstandardized coefficients (Table S3, S4, S5). In addition, the graphical representation of the flow‐flux relationship, presented on a runoff type basis is provided. [file JEQ2-54-450-s001.docx]

*Supplementary Table S1. Definition and Distribution of Categorical, Independent Variables*

| **Variable** | **Category** | **# of sites** | **# of events** |
| --- | --- | --- | --- |
| State | MN | 7 | 536 |
|  | WI | 15 | 803 |
|  |  |  |  |
| Soil type | Clay loam | 1 | 69 |
|  | Loam | 2 | 159 |
|  | Sandy loam | 1 | 31 |
|  | Silt loam | 17 | 970 |
|  | Silty clay loam | 1 | 110 |
|  |  |  |  |
| Hydrologic Group | A | 1 | 31 |
|  | B | 11 | 754 |
|  | C | 2 | 98 |
|  | C/D | 8 | 456 |
|  |  |  |  |
| Drainage class | Well drained, Moderately well drained | 17 | 1028 |
|  | Poorly drained, somewhat poorly drained | 5 | 311 |
|  |  |  |  |
| Manure | Yes | 19 | 1168 |
|  | No | 3 | 171 |
|  |  |  |  |
| Soil condition | Frozen | 22 | 549 |
|  | Non-frozen | 22 | 790 |
|  |  |  |  |
| Crop | Alfalfa | 7 | 237 |
|  | Corn | 18 | 632 |
|  | Corn and alfalfa | 3 | 181 |
|  | Pea | 1 | 12 |
|  | Soy | 11 | 270 |
|  |  |  |  |
| Tillage | Conventional Tillage (CT) | 13 | 794 |
|  | No Tillage (NT) | 5 | 331 |
|  | Reduced Tillage (RT) | 4 | 214 |
|  |  |  |  |
| Surface condition | Tilled – Crop | 17 | 427 |
|  | Tilled – No Cover | 13 | 407 |
|  | Tilled – Residue | 7 | 174 |
|  | No Till – Crop | 5 | 62 |
|  | No Till – No Cover | 4 | 135 |
|  | No Till – Residue | 5 | 134 |
|  |  |  |  |
| Water year | 2004 | 6 | 91 |
|  | 2005 | 7 | 74 |
|  | 2006 | 7 | 36 |
|  | 2007 | 7 | 42 |
|  | 2008 | 6 | 96 |
|  | 2009 | 3 | 30 |
|  | 2010 | 6 | 26 |
|  | 2011 | 7 | 63 |
|  | 2012 | 8 | 64 |
|  | 2013 | 9 | 108 |
|  | 2014 | 10 | 152 |
|  | 2015 | 12 | 92 |
|  | 2016 | 12 | 144 |
|  | 2017 | 13 | 154 |
|  | 2018 | 7 | 74 |
|  | 2019 | 7 | 93 |
|  |  |  |  |
| Water month | 1 - October | 15 | 32 |
|  | 2 - November | 5 | 12 |
|  | 3 - December | 20 | 28 |
|  | 4 – January | 21 | 76 |
|  | 5 - February | 21 | 137 |
|  | 6 - March | 22 | 282 |
|  | 7 - April | 18 | 134 |
|  | 8 - May | 20 | 175 |
|  | 9 - June | 22 | 208 |
|  | 10 - July | 18 | 110 |
|  | 11 - August | 21 | 76 |
|  | 12 – September | 16 | 70 |
|  |  |  |  |
| Runoff type | Rainfall on frozen ground | 22 | 230 |
|  | Rainfall on non-frozen ground | 22 | 790 |
|  | Snowmelt | 22 | 317 |

*Supplementary Table S2. Univariate statistics for runoff and sediment load (kg ha^-1^) and flow-weighted mean concentrations (FWMC) (mg L^-1^).*

|  | Runoff | Sediment | Sediment |
| --- | --- | --- | --- |
|  | mm | kg ha^-1^ | mg L^-1^ |
| n | 1339 | 1339 | 1339 |
| Minimum | 0 | 0 | 0 |
| 1^st^ Quartile | 0.49 | 0.31 | 25 |
| Mean | 5.83 | 38.72 | 832.07 |
| Median | 1.98 | 1.86 | 110 |
| 3^rd^ Quartile | 6.68 | 12.69 | 577.64 |
| Maximum | 99.5 | 2630 | 29310 |
| Std. Dev | 9.84 | 150.51 | 2299.46 |
| CV | 1.69 | 3.89 | 2.76 |
| Skewness | 3.57 | 8.65 | 6.53 |
| Kurtosis | 17.75 | 103.66 | 56.82 |

*Supplementary Table S3. Output of linear mixed model one (unstandardized coefficients) on TP FWMC, DP FWMC, TP, and DP (n=1318) (all data). All outcome variables were log-transformed. Marginal R^2^, describes variance from the fixed factors alone, and Conditional R^2^ describes variance from both fixed and random factors. Site and month were random factors. Manure application was coded as binary (0= manure applied, 1= no manure applied). Intercept represents a site in hydrologic group A, planted with corn with an active crop growing, in non-frozen conditions. Bolded values represent coefficients significant at p < 0.05 and italicized values represent coefficients significant at p < 0.1.*

| Variable | TP | DP | TP FWMC | DP FWMC |
| --- | --- | --- | --- | --- |
| Intercept | **-1.12±0.28** | **-1.79±0.24** | **0.49 ± 0.22** | -0.23 ±0.27 |
| Hydrologic group B | **-0.63±0.24** | *-0.40±0.21* | **-0.42 ± 0.19** | -0.23 ±0.23 |
| Hydrologic group C | -0.47±0.48 | -0.40±0.43 | -0.43 ± 0.38 | -0.26 ±0.47 |
| Hydrologic group C/D | **-0.67±0.232** | **-0.44±0.21** | **-0.51 ± 0.18** | -0.37 ±0.22 |
| Alfalfa | **-0.32±0.07** | **-0.14±0.06** | **-0.23 ± 0.05** | -0.08 ±0.05 |
| Corn and alfalfa | 0.20±0.23 | 0.21±0.20 | 0.09 ± 0.18 | 0.14 ±0.23 |
| Pea | -0.05±0.20 | *0.33±0.17* | -0.18 ± 0.13 | *0.27 ±0.14* |
| Soy | 0.01±0.05 | 0.02±0.04 | **-0.16 ± 0.03** | **-0.12 ±0.04** |
| No Till | 0.08±0.22 | **0.47±0.20** | 0.13 ± 0.17 | **0.58±0.21** |
| Reduced Tillage | -0.10±0.3 | 0.32±0.27 | -0.13 ± 0.24 | 0.23 ±0.29 |
| No Cover | **0.18±0.06** | **0.11±0.05** | **0.22±0.04** | **0.15±0.04** |
| Residue | **0.16±0.07** | **0.15±0.05** | **0.18 ± 0.04** | **0.18 ±0.05** |
| Manure application | 0.06±0.27 | 0.17±0.23 | -0.10 ± 0.21 | -0.04 ±0.26 |
| STP 0-15cm | 0 | 0.01±0.004 | **0.01±0.003** | **0.01±0.004** |
| STP 0-5cm | -0.002±0.003 | *-0.005±0.003* | 0.002±0.003 | -0.01±0.003 |
| Snowmelt | -0.01±0.07 | *0.14±0.05* | -0.04 ± 0.05 | **0.14 ±0.05** |
| Rainfall on frozen ground | **0.53±0.07** | *0.61±0.058* | -0.02 ± 0.05 | **0.09 ±0.05** |
| Marginal R^2^ | 0.17 | 0.23 | 0.17 | 0.25 |
| Conditional R^2^ | 0.27 | 0.30 | 0.32 | 0.38 |

*Supplementary Table S4. Output of linear mixed model two (unstandardized coefficients) on TP FWMC, DP FWMC, TP, and DP (n=1040). All the outcome variables in the table were log-transformed. Marginal R^2^, describes variance from the fixed factors alone, and Conditional R^2^, describes variance from both fixed and random factors, reported for each response variable. Site and month were random factors. Intercept represents a site in hydrologic group A, planted with corn with an active crop growing, in nonfrozen conditions. Bolded values represent p < 0.05. Italicized values represent p < 0.1.*

| Variable | TP | DP | TP FWMC | DP FWMC |
| --- | --- | --- | --- | --- |
| Intercept | **-0.78±0.22** | **-1.37±0.22** | **0.58±0.13** | -0.05±0.22 |
| Hydrologic group B | **-0.69±0.18** | **-0.42±0.18** | **-0.48±0.10** | -0.23±0.18 |
| Hydrologic group C | -0.42±0.37 | -0.30±0.38 | -0.31 ±0.2 | -0.05±0.39 |
| Hydrologic group C/D | **-0.62±0.17** | **-0.42±0.17** | **-0.46 ±0.1** | *-0.36±0.18* |
| Alfalfa | -0.12±0.08 | -0.05±0.07 | -0.06 ±0.05 | -0.04±0.06 |
| Corn and alfalfa | 0.18±0.16 | 0.19±0.17 | 0.09 ±0.08 | 0.13±0.18 |
| Pea | 0.01±0.19 | *0.31±0.17* | -0.08 ±0.14 | *0.26±0.15* |
| Soy | -0.07±0.06 | -0.04±0.05 | **-0.21 ±0.04** | **-0.15±0.04** |
| No Till | 0.24±0.17 | **0.66±0.17** | **0.27 ±0.10** | **0.76±0.18** |
| Reduced Tillage | -0.25±0.25 | 0.17±0.25 | **-0.36 ±0.13** | -0.05±0.26 |
| No Cover | *0.11±0.06* | *0.10±0.05* | **0.14 ±0.05** | **0.12±0.05** |
| Residue | 0.06±0.07 | 0.10±0.06 | *0.10 ±0.04* | **0.14±0.05** |
| Days since manure application | 0 | 0 | 0 | 0 |
| STP 0-15cm | 0 | *0* | **0** | 0.01±0.003 |
| STP 0-5cm | 0 | **-0.01±0.003** | 0 | **-0.01±0.003** |
| Snowmelt | -0.08±0.08 | 0.10±0.07 | -0.06 ±0.06 | **0.15±0.06** |
| Rainfall on frozen soil | **0.48±0.09** | **0.59±0.07** | -0.05 ±0.06 | 0.08±0.06 |
| Marginal R^2^ | 0.21 | 0.29 | 0.21 | 0.32 |
| Conditional R^2^ | 0.30 | 0.36 | 0.31 | 0.39 |

*Supplementary Table S5. Output of linear mixed model three (unstandardized coefficients) on TP FWMC, DP FWMC, TP, and DP (n=973). All the outcome variables in the table were log-transformed. Marginal R^2^ describes variance from the fixed factors alone, and Conditional R^2^ describes variance from both fixed and random factors, reported for each response variable. Site and month were random factors. Manure application was coded as binary (0= manure applied, 1= no manure applied); Soil condition was coded as binary (0= non-frozen soil; 1= frozen soil). Intercept represents a site in hydrologic group A, planted with corn with an active crop growing, in frozen conditions. Bolded values represent p < 0.05. Italicized values represent p < 0.1.*

| Variable | TP | DP | TP FWMC | DP FWMC |
| --- | --- | --- | --- | --- |
| Intercept | **-0.96±0.27** | **-1.50±0.23** | **0.44±0.21** | -0.10±0.27 |
| Hydrologic group B | **-0.61±0.22** | *-0.37±0.19* | **-0.42±0.17** | -0.22±0.23 |
| Hydrologic group C | -0.37±0.45 | -0.32±0.39 | -0.32±0.36 | -0.13±0.48 |
| Hydrologic group C/D | **-0.62±0.22** | *-0.36±0.19* | **-0.53±0.17** | -0.35±0.22 |
| Alfalfa | **-0.38±0.08** | -0.13±0.07 | **-0.34±0.05** | -0.09±0.06 |
| Corn and alfalfa | 0.25±0.21 | 0.26±0.18 | 0.18±0.17 | 0.23±0.23 |
| Pea | -0.27±0.19 | 0.14±0.16 | *-0.24±0.13* | 0.20±0.14 |
| Soy | -0.06±0.05 | -0.04±0.05 | **-0.19±0.04** | **-0.16±0.04** |
| No Till | 0.05±0.21 | **0.55±0.18** | 0 | **0.58±0.21** |
| Reduced Tillage | -0.27±0.28 | 0.26±0.24 | -0.32±0.22 | 0.12±0.3 |
| No Cover | **0.19±0.06** | *0.10±0.05* | **0.22±0.04** | **0.11±0.04** |
| Residue | **0.16±0.07** | **0.16±0.06** | **0.22±0.05** | **0.21±0.05** |
| Manure application | -0.12±0.24 | 0.27±0.24 | -0.15±0.2 | -0.08±0.26 |
| Nonfrozen soil | **-0.76±0.08** | **-0.85±0.06** | 0.08±0.05 | *-0.10±0.06* |
| STP 0-15cm | 0 | *0.1±0.003* | **0.01±0.003** | **0.01±0.004** |
| STP 0-5cm | 0 | **-0.01±0.003** | 0 | *0* |
| Precipitation | **0.01±0.001** | **0.01±0.001** | 0 | 0 |
| Storm duration | 0.004±0.004 | 0.01±0.003 | 0 | 0 |
| Average Intensity | -0.08±0.07 | -0.05±0.06 | -0.06±0.05 | -0.01±0.05 |
| 5 Min Max Intensity | **0.09±0.02** | **0.03±0.02** | **-0.06±0.01** | *0* |
| Antecedent Rainfall 2 D | **0.12±0.03** | **0.11±0.02** | -0.03±0.02 | -0.01±0.02 |
| Marginal R^2^ | 0.33 | 0.38 | 0.26 | 0.29 |
| Conditional R^2^ | 0.42 | 0.45 | 0.39 | 0.44 |

*Supplementary Figure 1. Relationships between log-transformed total phosphorus load (TP) and dissolved phosphorus load (DP), and runoff. Snowmelt on frozen ground (n = 317), rainfall on frozen ground, (n=232), and rainfall on non-frozen ground (n=790) are shown. Linear regression models and R^2^ were reported for each figure.*
